# SimFuse: A Novel Fusion Simulator for RNA Sequencing (RNA-Seq) Data

**DOI:** 10.1155/2015/780519

**Published:** 2015-12-29

**Authors:** Yuxiang Tan, Yann Tambouret, Stefano Monti

**Affiliations:** ^1^Bioinformatics, Boston University, Boston, MA 02215, USA; ^2^Research Computing Services (IS&T), Boston University, Boston, MA 02215, USA; ^3^Section of Computational Biomedicine, Boston University, MA 02215, USA

## Abstract

The performance evaluation of fusion detection algorithms from high-throughput sequencing data crucially relies on the availability of data with known positive and negative cases of gene rearrangements. The use of simulated data circumvents some shortcomings of real data by generation of an unlimited number of true and false positive events, and the consequent robust estimation of accuracy measures, such as precision and recall. Although a few simulated fusion datasets from RNA Sequencing (RNA-Seq) are available, they are of limited sample size. This makes it difficult to systematically evaluate the performance of RNA-Seq based fusion-detection algorithms. Here, we present SimFuse to address this problem. SimFuse utilizes real sequencing data as the fusions' background to closely approximate the distribution of reads from a real sequencing library and uses a reference genome as the template from which to simulate fusions' supporting reads. To assess the supporting read-specific performance, SimFuse generates multiple datasets with various numbers of fusion supporting reads. Compared to an extant simulated dataset, SimFuse gives users control over the supporting read features and the sample size of the simulated library, based on which the performance metrics needed for the validation and comparison of alternative fusion-detection algorithms can be rigorously estimated.

## 1. Introduction

A gene fusion, also referred to as chromosomal translocation, denotes the event whereby two normally separated genes are joined together as a consequence of a genomic rearrangement following DNA replication. Gene fusions are known to play an important role in tumorigenesis in nearly all tumor types [1, 2]. Because RNA Sequencing (RNA-Seq) provides high coverage and reveals expressed gene fusion transcripts, RNA-Seq based fusion-detection is a standard component of functional cancer genomic research.

Currently, there are more than 15 RNA-Seq based fusion-detection tools published [3–9]. Most of these tools were tested only on real data (cell-lines or patient samples) with RT-PCR validations of a limited number of predicted fusions, which precluded the accurate estimation of recall and precision. Therefore, real data are useful to test whether a method is successful in detecting specific events but are not sufficient to comprehensively estimate the method's predictive performance. A statistically powerful simulated dataset containing large numbers of known true positives and true negatives is the complementary solution.

Some of the published fusion-detection tools [9–11] utilized an RNA-Seq based simulated fusion dataset generated as part of the evaluation of FusionMap [5]. However, this is a small dataset representing a single sample and a total of 50 fusions. A fusion simulator able to automatically generate multiple datasets with various numbers and types of fusion supporting reads could facilitate the performance evaluation of new and existing fusion-detection algorithms. Here, we propose SimFuse, a novel fusion simulator, to address this gap.

SimFuse uses real data to generate background reads. SimFuse can generate multiple fusion events with different numbers and types of supporting reads. With sufficient sampling, users can minimize random effects and accurately estimate the performance of a fusion-detection algorithm. Additionally, with SimFuse generated data, users can precisely estimate fusion-detection recall and precision rates as a function of the numbers or types of supporting reads. Availability: SimFuse is free for noncommercial use at (https://github.com/yuxiangtan/SimFuse).

## 2. Methods

**Figure 1:**
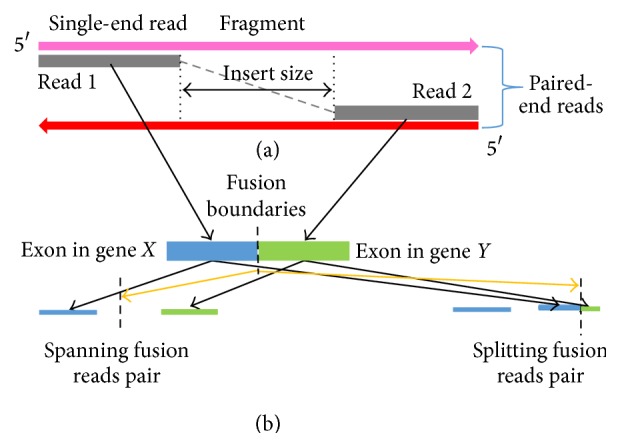
Definition of essential terms. (a) Definition of fragment, read, and insert size. A fragment is sequenced in both directions to get a pair of reads in the paired-end sequencing. The size of the nonsequenced portion between the paired ends is called insert size. (b) Definition of spanning read and splitting read pairs. The fusion boundaries are in the insert region in a spanning read pair, while the fusion boundaries fall within one of the reads in a splitting read pair.

We begin by defining some essential terms used throughout the paper (Figure 1). We define a* fragment* as a contiguous sequence of nucleotides from a cDNA molecule. The distribution of fragment lengths should approximately follow a Gaussian distribution. We define a* read* as the sequenced end of a fragment. We define* pair-end sequencing* as the procedure of sequencing both ends of the same fragment, and the two sequenced ends as* paired ends*. We define* insert-size* as the size of the nonsequenced fragment portion between the paired ends. We define* fusion boundaries* as the precise, nucleotide-level genomic breakpoint coordinates on both sides of the fusion gene pair. Lastly, we define* spanning* reads as those reads that have the fusion boundaries in the gap between the paired ends. Conversely, a* splitting* read has the fusion boundaries within one of the paired-end reads.

**Figure 2:**
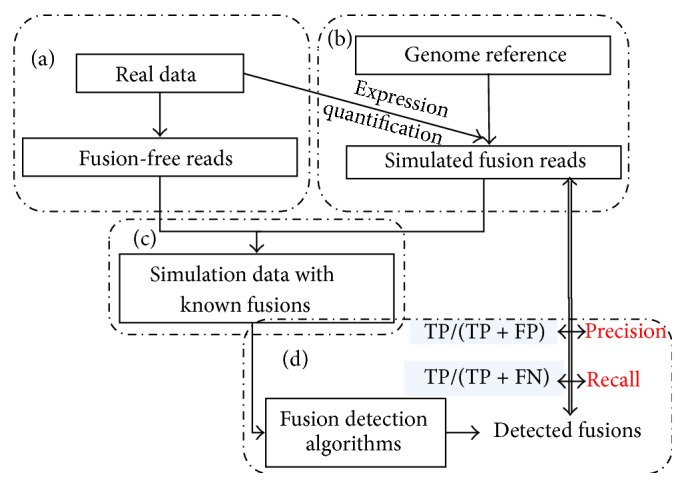
SimFuse workflow. (a) Fusion free reads are extracted from the real data. (b) The expression quantification from real data and a genome reference are used to simulate fusion reads. (c) Fusion-free reads and simulated fusion reads are merged. (d) Running a fusion-detection algorithm on the simulation dataset generates results of detected fusions. Comparison of these results with the list of simulated fusions yields fusion-detection performance estimates (recall and precision) for the algorithm.

**Figure 3:**
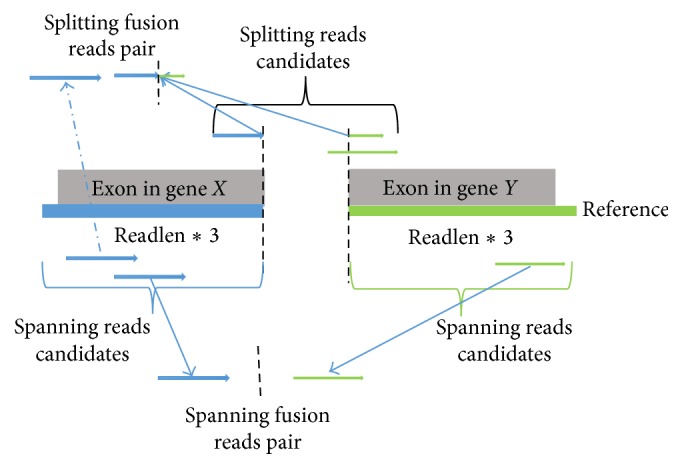
Workflow of generating fusion supporting reads. An exon in gene *X* and an exon in gene *Y* are randomly selected. Exon boundaries are used as the fusion boundaries. Spanning reads are randomly generated from within a prespecified range (read-length *∗* 3 by default) from the boundaries. To generate a splitting read, two fragments from the two genes *X* and *Y* are generated, with their sum length being equal to the required read-length. A spanning read is randomly generated from either of the two exons to match the splitting read into a splitting read pair.

The input to SimFuse consists of a pair-end aligned BAM file with known read length and distribution of fragment lengths (the insert-size must be positive in order to simulate spanning reads following this distribution). This BAM file can be the pure SAM/BAM output from aligners without additional filtering. SimFuse consists of the following four modules (Figure 2):

(1) *Extraction of Fusion-Free Reads (from the Input BAM File, to Build the Background Read Distribution).* Generally, a real dataset from patients or cell-lines is preferred. The benefit of using real data to generate the background is that it mimics the background noise in real data and also captures the realistic variation in gene expression levels. To filter all the potential fusion-supporting reads from the real data, only the pair-end aligned reads consistent with the distribution of fragment lengths are kept.
(2) *Simulation of Fusion Supporting Reads*. To fully control the simulation, SimFuse uses a genome reference (e.g., hg19.fa) as the template to generate supporting reads for simulated fusions (Figure 3). First, genes are binned into *M* expression subgroups based on the corresponding number of raw reads in the input data. In the default setting, raw read counts are grouped into the *M* ranges [0 ~ 10), [10 ~ 10^2^), [10^2^ ~ 10^3^),…, [10^*M*−1^ ~ 10^*M*^]. For each expression subgroup, *N* (the number of fusions to be simulated in a group; *N* = 100 as default) genes are randomly picked without replacement as the fusion genes. For each fusion gene, a fusion gene partner is randomly selected from each of the expression subgroups. For each pair of genes, splitting and spanning read pairs are simulated by using the reference sequence of these genes (see supplementary material in Supplementary Material available online at http://dx.doi.org/10.1155/2015/780519). At the end of this process, a total of *M* × *N* × *M* fusion events are generated, with *N* fusion events for each of the *M* × *M* combinations of expression groups. Finally, all the simulated reads are merged with the background reads into a newly created FASTQ file, and a list of fusion-gene pairs is generated.
(3) *Wrapper for the Automatic Simulation of Multiple Datasets* (*with Different Supporting Reads).* To fully estimate the performance of a fusion-detection algorithm, a simulated dataset with large enough sample size is needed. Additionally, the performance of a fusion-detection algorithm may be dependent on the number and the types (i.e., spanning or splitting) of supporting reads. To be user friendly, SimFuse has a wrapper function that simultaneously generates *K* simulations with *L* different combinations of supporting read numbers, which yields a total of *M* × *N* × *M* × *L* × *K* fusion events.
(4) *Generation of a Summary of Fusion-Detection Results* (*from a Given Algorithm for the Whole Simulation).* To compare fusion-detection results from different algorithms efficiently, we convert these results to a uniform format and then use a SimFuse function for summarization (see supplementary Table  1 and Figure  S1).

To estimate the performance of a fusion-detection algorithm, we use recall and precision rates instead of sensitivity and specificity [12]. Precision is more informative than specificity, since in the fusion-detection problem the number of true negatives (TN) is always much larger (~20 K genes in the human genome) than the number of true positives (TP). As a result, unless the number of false positives (type I errors) is extremely large, the specificity will always be close to 1 and will not adequately capture the difference among competing detection algorithms:

  recall: true positive/(true positive + false negative),
  precision: true positive/(true positive + false positive).

## 3. Results

### 3.1. A Simulation Example from SimFuse

We used an ENCODE MCF-7 cell-line dataset (SRR521521) to extract the background reads to be included in the simulation. This dataset consists of 76 bp pair-end reads, with a median fragment size of 192 bp and a standard deviation of 29 bp. Accordingly, we used a splitting-to-spanning read ratio of 19 : 5 for the generation of the fusion supporting reads (see supplementary materials). We next generated *K* = 100 independent simulations, and, for each simulation, we generated *L* = 10 combinations of supporting read numbers, ranging from 1 splitting read and no spanning reads (1 : 0) to 100 splitting reads and 26 spanning reads (100 : 26), with *N* = 100 fusion events for each combination (see supplementary Table  2). Grouping based on expression levels is not considered and we set *M* = 1, since the data already has 100,000 (1 × 100 × 1 × 10 × 100) fusions. The entire data generation procedure was run on a 16-core 2.3 GHz AMD Opteron 6276 machine with 64 GB memory. Because SimFuse does not currently support parallel processing, it took 18.5 hours to complete this task using a single core.

**Figure 4:**
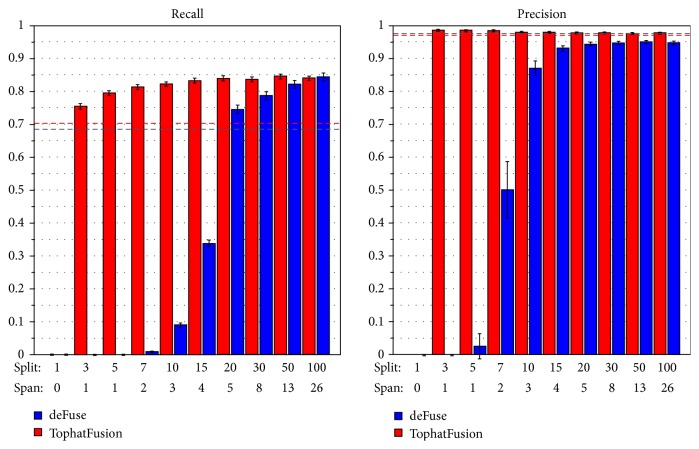
Barplot of recall and precision rates for deFuse and TophatFusion. Blue bars indicate deFuse results and red bars indicate TophatFusion results. The *x*-axis is indexed by the supporting read groups, with each group corresponding to the indicated splitting-to-spanning read ratio. The *y*-axis reports precision and recall rates. The red dash line shows estimates for TophatFusion from the FusionMap dataset, while the blue dash line shows estimates for deFuse.

We used deFuse [3] and TophatFusion [8] as the two fusion-detection algorithms to analyze the simulated datasets, with default parameter settings (see supplementary materials).  Figure 4 summarized the recall and precision rates of the two algorithms. The recall rate of deFuse was lower than TophatFusion in the low supporting read range but increased and surpassed the recall rate of TophatFusion in the detection of fusions with at least 100 splitting and 26 spanning reads. Conversely, TophatFusion achieved a 75% recall rate with as few as 3 splitting and 1 spanning reads. However, it plateaued at a lower 84% recall rate with 20 splitting and 5 spanning reads. The precision of deFuse was relatively low in the low supporting read range but reached a maximum of 95% when detecting fusions with at least 20 splitting and 5 spanning reads. The precision of TophatFusion was always high but slightly decreased as the number of supporting reads increased. The likely explanation for this phenomenon is that as the number of supporting reads increases, the chance of detecting multiple alignment events also increases.

### 3.2. Comparison with Existing Simulated Data

The simulated dataset from FusionMap is the most accessible and popular simulated RNA-Seq fusion dataset currently available [5, 11]. Hence, we aimed to evaluate our SimFuse-based comparison of deFuse and TophatFusion against the analogous comparison based on the FusionMap simulated dataset. To make the comparison fair, deFuse and TophatFusion were run with the same parameter settings used with the SimFuse datasets. On the other hand, because the FusionMap dataset has only one sample with 50 fusions and no replicates, it was not possible to estimate the corresponding distributions of recall and precision rates. The overall recall rate was 68% (34/50) for deFuse and 70% (35/50) for TophatFusion, and the overall precision rate was 97.1% (34/35) for deFuse and 97.2% (35/36) for TophatFusion. These estimates from the FusionMap dataset are concordant with those from the SimFuse datasets. Additionally, in these 50 fusions, the supporting read numbers range from 2 to 1587, which makes it not possible to rigorously estimate recall and precision rates at different supporting read levels. Nevertheless, the analysis on the FusionMap dataset revealed that deFuse could not detect fusions with fewer than 10 reads while TophatFusion could, thus confirming the more detailed conclusions drawn from the SimFuse datasets.

## 4. Discussion

We have presented a new fusion simulator, SimFuse, for the evaluation and comparison of fusion-detection algorithms from RNAseq data. To our knowledge, this is the first publicly available tool for the simulation of RNAseq libraries enriched for fusion events. The simulator's main selling point is its capability of generating large numbers of fusion events with customizable characteristics, including number of supporting reads and ratio of splitting-to-spanning reads, among others. An additional advantage is SimFuse's ability to simultaneously generate a large number of samples, providing great statistical power for fusion-detection algorithm performance estimation. The capability of generating fusions with a tunable number of supporting reads also allows users to estimate the minimum number of reads necessary to achieve desired rates of fusion-detection recall and precision. Moreover, SimFuse provides detailed individual supporting read information for each simulated fusion, and as a result, users can evaluate the fusion-detection algorithm performance on every single supporting read. This is a unique feature that no other simulated RNA-Seq fusion dataset provides.

We used SimFuse to generate a large dataset of fusion-rich samples, which was used to compare two state-of-the-art fusion-detection tools, TophatFusion [8] and deFuse [3]. By comparing the number of known fusion-supporting reads in the SimFuse dataset with those identified by deFuse and TophatFusion, we found that deFuse and TophatFusion could detect splitting and spanning reads well in most cases. However, in some cases, these two methods reported more supporting reads than the actual number of simulated reads, which might suggest a propensity of these algorithms to report false positives.

SimFuse already allows for the control of fusion parameters (such as number of supporting reads and ratio of splitting-to-spanning reads) and relies on a reference genome template for the generation of the fusion supporting reads, but additional parameters will be included in future improvements to more closely approximate real data. For example, we can introduce tunable mutation rates for each base in the template genome reference, or relax the location requirement of fusion boundaries, or offer the option of alternative splicing conjunction between exons.

SimFuse is available as open source software on github, and input from any developers is welcome. Our hope is that this package will provide for a starting point and that additional functions and improvements will be contributed by the community.

## Supplementary Material

In this supplementary, we descript the four main sessions of SimFuse in details (including versions of tools used). We also provide the parameters used for running deFuse and TophatFusion for the comparision. Supplementary tables, which are complementary to figures of barplots are included. The logic of the summary function is presented in the supplementary figure.
