# Supplementary material for: SimFuse: A Novel Fusion Simulator for RNA Sequencing (RNA-Seq) Data
Source: Biomed Res Int. 2015 Dec 29;2015:780519. doi: 10.1155/2015/780519 (PMC4709598; doi:10.1155/2015/780519)
Supplement: Supplementary file 1 — In this supplementary, we descript the four main sessions of SimFuse in details (including versions of tools used). We also provide the parameters used for running deFuse and TophatFusion for the comparision. Supplementary tables, which are complementary to figures of barplots are included. The logic of the summary function is presented in the supplementary figure. [file 780519.f1.pptx]

## Slide 1
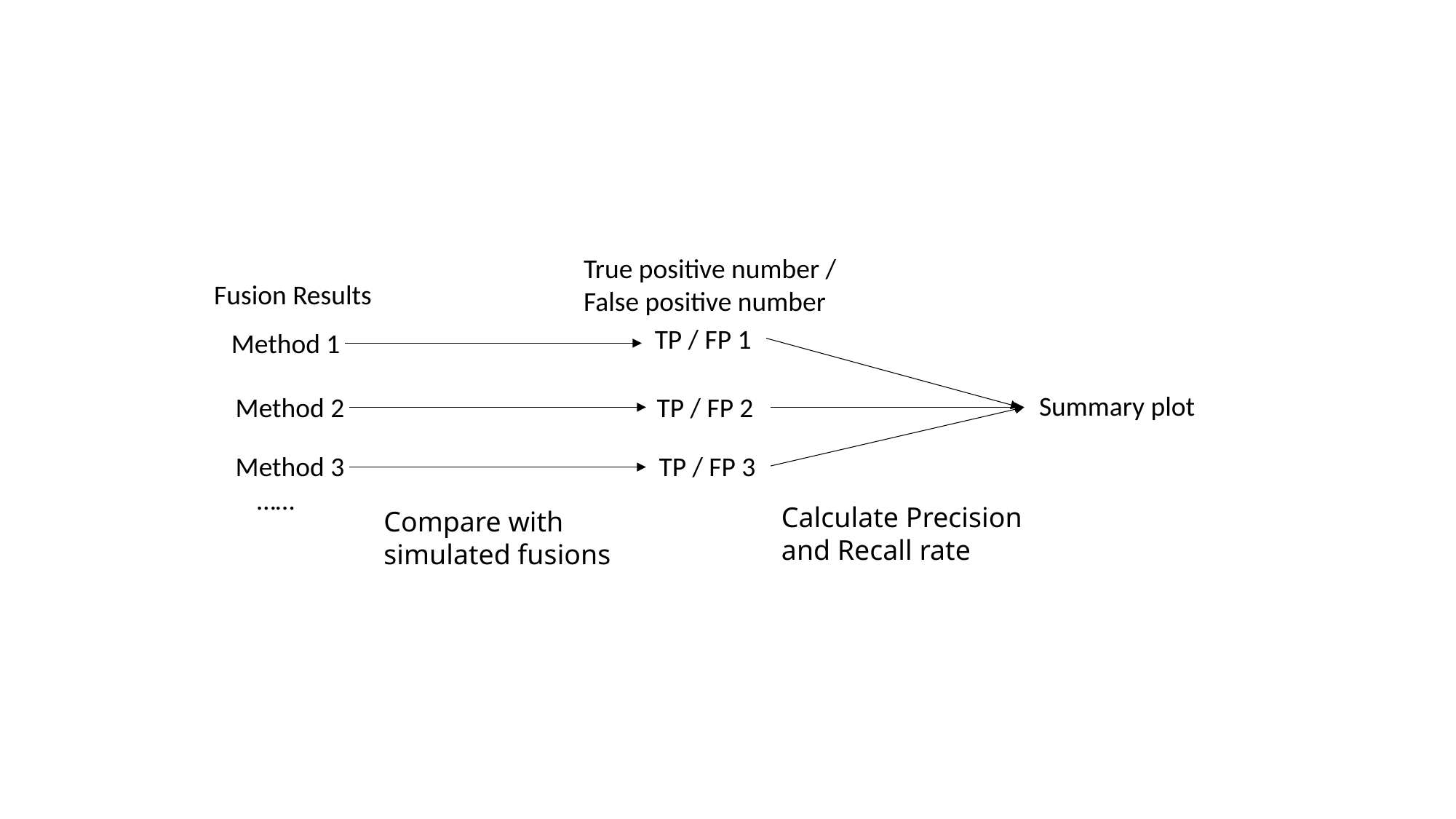

True positive number / False positive number
Fusion Results
TP / FP 1
Method 1
Summary plot
Method 2
TP / FP 2
Method 3
TP / FP 3
……
Calculate Precision and Recall rate
Compare with simulated fusions
